# Evaluating the effects of an intervention to improve the health environment for mothers and children in health centres (BECEYA) in Mali: a qualitative study

**DOI:** 10.11604/pamj.2023.44.138.36736

**Published:** 2023-03-17

**Authors:** Patrice Ngangue, Katherine Robert, Birama Apho Ly, Fatoumata Traoré, Leonel Philibert, Maude Vezina, Nestor Bationo

**Affiliations:** 1 Faculté des Sciences Infirmières, Université Laval, Quebec, Canada,; 2 Faculté de Médecine, Université Laval, Quebec, Canada,; 3 University of Sciences, Techniques and Technology of Bamako, Bamako, Mali,; 4 Faculty of Health Sciences, University of Ottawa, Ottawa, Canada,; 5 Unité d'Enseignement et de Recherche en Sciences de la Santé, Université Joseph Ki-Zerbo, Ouagadougou, Burkina Faso

**Keywords:** Community health centres, hygiene, infection prevention, qualitative study, Mali

## Abstract

**Introduction:**

an intervention aiming to improve the maternal and children environment in healthcare facilities (BECEYA) was launched in three regions of Mali. This study aimed to explore the perceptions and experiences of patients and their companions, community actors, and healthcare facilities staff on the effects of the BECEYA intervention in two regions of Mali.

**Methods:**

we conducted a qualitative study using an empirical phenomenological approach. Through purposive sampling, women who attended antenatal care in the selected healthcare centres, companions, and health facility staff members were recruited. Data were collected during January and February 2020 through semi-structured individual interviews and focus groups. According to Braun and Clarke's approach, audio recordings were transcribed verbatim, and a thematic analysis was conducted in five main steps. Donabedian conceptual framework of quality of care was used to present the perceived changes following the implementation of the BECEYA project.

**Results:**

we recruited 26 participants in individual interviews (20 women attending prenatal care and maternity services, 10 per health centre, four companions, and two healthcare centre managers) and 21 healthcare centre staff members (10 in Babala, 11 in Wayerma 2) in focus groups. Themes that emerged from data analysis are perceived changes in terms of infrastructure (perceived changes in the characteristics of the healthcare facilities setting, including the infrastructure introduced by the BECEYA project), process (changes in the delivery and use of care introduced or resulting from BECEYA activities), and outcome (the direct and indirect effects of these changes on the health status of patients and the population).

**Conclusion:**

the study highlighted some positive effects on women users of the services, their companions, and health centre staff following the implementation of the intervention. This study contributes to showing some links between improving the environment of healthcare centres and the quality of care in developing countries.

## Introduction

Ensuring access to water, sanitation, and hygiene (WASH) in health facilities is part of the United Nations' agenda for global transformation through the Sustainable Development Goals [1,2]. In addition, essential WASH services in health facilities and maternity wards are fundamental to providing quality care and fulfilling primary healthcare commitments [3,4]. In healthcare facilities (HCFs), WASH interventions promote a healthy environment to minimize disease risk for users (patients, healthcare staff, and maintenance staff) and the surrounding community. Interventions include providing a water point and improving the sanitary environment (including sanitation, availability of hand hygiene and infection control facilities, biomedical waste management, and cleaning the physical environment) [3,4]. Sub-Saharan Africa has the lowest access to safe water and sanitation initiatives and the lowest rate of improvement in sanitation, with an estimated 695 million people still using the unimproved infrastructure [1,2]. Mali, a continental country in West Africa, has minimal improvement in WASH. Indeed, in Mali, 50% of facilities need more water storage, and over 70% have poor-quality water. In addition, there are no national policies and plans on WASH in healthcare facilities [5]. In 2015, an intervention aiming to improve the maternal and children environment in healthcare facilities (BECEYA) was launched in three regions of Mali. The project was a collaboration between the Government of Canada and the Mali Ministry of Health and Social Affairs. BECEYA intervention components include the management of hygiene and biomedical waste; improving selected health facilities infrastructures mainly through four essential services: water supply (access points to drinking water, washing areas, shower); installation/rehabilitation of adapted latrines; and solar energy electrification. As part of these essential components, the intervention aimed to reinforce the medical staff's capacity to play their role in biomedical waste management through support to the national and regional health and public hygiene directorates. In addition, the implementation of this intervention involved the participation of various community actors, such as community health and women's associations. This study aimed to explore the perceptions and experiences of patients and their companions, community actors, and healthcare facilities staff on the effects of the BECEYA intervention in two regions of Mali.

## Methods

**Study design and setting:** we conducted a qualitative study through individual semi-structured interviews and focus groups. Qualitative studies are indicated in research on social phenomena that are difficult to quantify [6]. Therefore, an empirical phenomenological approach was used to obtain detailed descriptions of perceptions and experiences of patients and their companions, community actors, and healthcare facilities staff on the effects of the BECEYA intervention. Phenomenological research describes commonalities of participants' experiences across a population [7]. The study was conducted in two Malian regions (Kayes and Sikasso) and supported by the BECEYA project. As a result, two health centers were selected (Babala and Wayerma 2).

**Data collection:** data were collected during January and February 2020 through semi-structured individual interviews and focus groups. Focus group interviews were conducted with the health centers' staff concerned. In addition, semi-structured, individual interviews were conducted with women attending antenatal care and their companions, the manager of the healthcare center. The individual semi-structured interviews and focus group guides were developed based on Donabedian's conceptual framework adapted to the context of the BECEYA project [8]. Interview guides were translated into the local language (Bambara) and tested with a population sample. With participant permission, all interviews were audio-recorded. Sociodemographic characteristics were collected at the start of the interview, followed by questions about participants' experiences and perceived effects of the BECEYA project.

**Data analysis:** the audio recordings were transcribed verbatim, translated into French, and reviewed by the interviewers for accuracy. All authors agreed and chose the highlighted quotations during the data analysis. All quotes were translated into English by Patrice Ngangue. Analyses were carried out using the qualitative data analysis (QDA) miner qualitative analysis software from Provalis. A thematic analysis was conducted in five main steps: i) reading and familiarizing with the data; ii) developing initial codes (coding); iii) searching for themes; iv) reviewing potential themes, and v) identifying categories of themes using a mixed (inductive and deductive) approach [9,10]. Two research team members (Katherine Robert and Maude Vezina) performed each step. Throughout this study, we followed the consolidated criteria for reporting qualitative research (COREQ) [11].

**Ethical considerations:** ethics approval for this study was obtained from the Institutional Ethics Committee of the Faculty of Medicine, Odontostomatology and Pharmacy of the University of Sciences, Techniques and Technologies of Bamako, Mali (N° 2020/06/CE/FMOS/FAPH). An information sheet explaining the research objectives was given and presented to each participant. Signed consent was required and obtained before each interview. Confidentiality was assured by using numbers instead of names and removing identifying information from the transcripts. All audio recordings and transcripts were saved on a password-protected computer.

## Results

Our sample consisted of 26 participants in individual interviews (20 women attending prenatal care and maternity services, 10 per health center, four companions, and two healthcare center managers) and 21 participants in focus groups (21 healthcare center staff members, 10 in Babala, 11 in Wayerma 2). Characteristics of women attending prenatal care and maternity services, companions, and healthcare staff are available in Table 1, Table 2 and Table 3. Donabedian's conceptual framework of quality of care 11 was used to present the perceived changes following the implementation of the BECEYA project. The themes are related to infrastructure (perceived changes in the characteristics of the healthcare facilities setting, including the infrastructure introduced by the BECEYA project), process (changes in the delivery and use of care introduced or resulting from BECEYA activities), and outcome (the direct and indirect effects of these changes on the health status of patients and the population).

**Table 1:** characteristics of women attending prenatal care and maternity services (n = 20)

	Babala	W2		Babala	W2
**Age (Years)**			**Occupation**		
15 - 20	4	2	Housewife	4	5
21 - 25	-	3	Trader	1	2
26 - 30	6	3	Teacher	-	1
31 - 35	-	1	Dressmaker	1	1
36 - 40	-	1	Nurse	-	1
			Farmer	3	-
			Student	1	-
**Total**	10	10	**Total**	10	10
**Place of residence**			**Place of delivery**		
Babala	9	-	Babala health centre	8	-
Wayerma 2	-	6	Wayerma 2 health centre	-	10
Other	1	4	Other health centre	2	-
**Total**	10	10	**Total**	10	10
**Matrimonial status**			**Number of prenatal visits**		
Married	10	9	4	10	9
Single	-	1	5		1
**Tota**l	10	10	**Total**	10	10
**Level of education**	<		Babala	Wayerma2	
None			5	3	
Primary school			4	3	
Secondary school			-	2	
University			-	2	
**Total**			10	10	

**Table 2:** characteristics of companions

	Babala	W2		Babala	W2
**Age (years)**			**Sex**		
15 - 20	1	-	Women	1	1
21 - 25	-	1	Men	1	1
26 - 30	-	1			
31 - 35	-				
36 - 40	-				
41 - 45	1		;		
**Total**	2	2	**Total**	2	2
**Place of residence**			**Occupation**		
Babala	1	-	Trader	-	1
Wayerma 2	-	1	Farmer	1	-
Other	1	1	Bricklayer	-	1
			Mechanic	1	-
**Total**	2	2	**Total**	2	2
**Matrimonial status**			**Family relationship**		
Married	1	1	Aunt	1	-
Single	1	1	Brother-in-law	1	1
			Mother-in-law	-	1
**Total**	2	2	**Total**	2	2

**Table 3:** characteristics of healthcare staff

Age (years)	Babala	W2	Occupation	Babala	W2
20 - 25	1	-	Matrons	3	1
26 - 30	2	1	Security guard	1	1
31 - 35	1	5	Manager	1	-
36 - 40	2	-	Assistant nurse	1	-
41 - 45	-	1	Cleaning agent	-	1
46 - 50	2	3	Nurses	-	6
51 - 55	1	-	Technologist	1	-
56 - 60	-	1	Midwife	2	1
61 - 65	1	-	Physicians	1	1
**Total**	10	11	**Total**	10	11
**Place of residence**			**Number of years on the job**		
Babala	8	-	0 - 5	3	6
Wayerma 2	-	5	6 - 10	3	1
Autre	2	6	11 - 15	2	-
			16 - 20	1	4
			21 - 25	1	-
**Total**	10	11	**Total**	10	11
**Matrimonial status**			Sex		
Married	10	11	Men	5	2
			Women	5	9
**Total**	10	11	**Total**	10	11

**Perceived changes in infrastructure from the perspective of women attending antenatal care and their companions:** the various changes in the infrastructure of the community health centers (CSCom) reported by the participants concern all the components of WASH, namely hygiene (provision of gender-specific latrines, new handwashing equipment, i.e. devices consisting of a bucket of drinking water above a wastewater collection container and a holder for soap, the addition of a washing area for clothes, medical instruments, dishes, etc.); water (new water supply facilities including a water tower and drinking water taps); sanitation (the installation of the incinerator and the equipment for the management of biomedical waste, including the three-colour bins). In addition, electricity has been installed in certain sections of the health center. The women users and their companions commented on their feelings about the changes brought about by these new infrastructures, particularly about the cleanliness of the health centers: “Before there was no maintenance in this center, but today there is much hygiene in this center, the latrines are cleaner too, every time I go into the latrines, I see that they are clean” (W2_Wo9). The changes in the infrastructure of the CSCom brought about by the BECEYA project have enabled women to give birth and professionals to work in a better-equipped environment. For example, Babala users reported that “before, women used to deliver on the table without spreading the plastic, but now he spreads the plastic on the table before delivering” (B_ Wo10), while in Wayerma 2, it is that “Now new deliveries lie under clean mosquito nets. Otherwise, there was no net before. So, women were only lying on beds without nets. The distribution of insecticide-treated mosquito nets in the health center is part of a major effort by the Ministry of Health to protect pregnant women and children aged 0-5 years from mosquito bites that could be dangerous for them.

**Perceived changes in infrastructure from the perspective of health center staff:** the health center's staff also reported their perceptions of the various changes following the BECEYA project's implementation. Their perceptions corroborate those of women attending antenatal care and their companions. They said that new infrastructures implemented by the BECEYA project in the health facilities improved the quality of services and care. For example, one participant revealed, “Before the BECEYA project, there was only one restroom for the whole health center. Thanks to BECEYA, the health center staff, patients, and even the disabled have their latrines (one for men and one for women)” (B_FG_HCP). Another participant appreciates the health center's hygiene and cleanliness: “Before the arrival of BECEYA, we put the waste in any way. Now, for every piece of rubbish in the center's courtyard, we pick it up and put it in the appropriate container. We teach women how to deal with waste. Instead of throwing it in the yard, we show them the appropriate container” (B_FG_HCP).

**Perceived changes in the care process from the perspective of women attending antenatal care and their companions:** the new infrastructure has led to changes in practices in the health facility. For example, women attending antenatal care and their companions were asked about the changes they had observed in their health center regarding patient care. They described changes in service users' care practices and behaviors, women in their communities, and staff. These changes result from the improved infrastructure at the two health centers. They reported the excellent reception and quality of care received and the perceived competence of the staff in the health center. These elements positively influence women's experience at the health center and encourage them to use the services, as reported by one user: “I appreciate the reception, when I came straight away a midwife welcomed me well, she took my papers and guided me to the consultation room, and I liked that a lot” (W2_Wo10). Another participant spoke of the quality of care, particularly the postpartum follow-up, which she said had improved: “Nowadays, when you give birth in the center, you stay under surveillance for six hours before leaving for home, which we didn't do before. Before BECEYA, some women left for home right after their delivery” (W2_Wo7).

**Process changes from the perspective of health center staff:** the changes observed and reported by the health staff affect access to care for the poorest and the supply of essential medicines and inputs. For example, the reduction in delivery costs is reported by one participant: “Delivery costs were prohibitive here, but this is not the case now” (B_ FG_HCP). In addition, health centers are better supplied with inputs to provide care to clients. This motivates clients to visit the health center more often, as staff members report in the following excerpts: “Mothers accompany their children to the Central Ohio Mental Health Center (ComHC) when they are ill. Medicines are available at the health center (W2_personnel 3). Participants also perceived changes in the women's involvement in sanitation at the health center. To illustrate this, one participant reported: “...they have changed, because every Saturday they come here to the center to wash our rooms, sweep the courtyard, and then every 15^th^ of the month, it is the village sanitation day...” (B_HCM).

**Perceived effects on health outcomes from the perspective of women attending antenatal care and their companions:** most participants perceived the positive impacts of the BECEYA project on the health of women and children. The project has increased awareness of the importance of attending health centers during pregnancy and favoring assisted childbirth. According to one respondent, “The changes I have observed are that absolute neutrophil count (ANC) is beneficial for women. Moreover, it makes childbirth easier” (B_Ac2). The awareness and participation of women in health-related activities have led to changes in the health of community members. In this regard, one user reported hygiene awareness and new care practices: “The application of advice provided by health workers has reduced several diseases; the application of bleach has also reduced diarrheal diseases” (B_Wo).

**Perceived effects on health outcomes from the perspective of the health center staff:** the health centers that benefited from the BECEYA project saw their activities increase, mainly thanks to the awareness-raising done in the community. As an illustration, one participant reports: “Sensitization has changed many things in the work, especially in prenatal consultations, vaccinations, deliveries, family planning, and postnatal consultations. With the arrival of BECEYA, these activities have expanded” (B_ FG_HCP). The BECEYA project has also had positive results in terms of gender relations. Men have realized the need to give their wives more freedom by being involved in awareness-raising activities. One participant reported that “There has been a change, as before men did not accept their wives to advocate or speak in public. However, this has changed since the arrival of BECEYA. The proof is that today the women of Babala have gone to another village to raise awareness, even some men accompany them because they have understood that it is a good thing” (B_ FG_HCP).

## Discussion

This study aimed to analyze the perceptions and experiences of service users and health staff on the effects of a project to improve the health environment of community health centers in two regions of Mali. The study results provide an overall picture of the changes observed in the health centers supported by the BECEYA project from the perspective of the women users of the services, their companions, and the health center staff. Changes repeatedly reported in both health centers were the environment's cleanliness, salubrity, and infrastructure improvement. These improvements were associated with an increase in the quality of services and work capacity. In addition, there was an unprecedented motivation among women to use health services and their commitment to maintaining cleanliness in their living environment. Overall, changes in infrastructures and processes positively impacted the health of patients and the whole community. Health facilities, water, hygiene, and sanitation (WASH) services help respond to outbreaks and prevent healthcare-associated infections. They are essential to ensure healthcare quality [5,12,13]. Barriers to the use of hospital care are mainly related to the quality of care, especially for maternity services (often inadequate, unaffordable, understaffed, and needing more medically trained professionals) [12,13]. In resource-limited countries, access to affordable energy and adequate clean water in health facilities is a significant factor in high maternal and infant morbidity and mortality [3,14-16]. Lack of electricity makes it impossible to operate cold chains capable of storing life-saving vaccines 16, while inadequate drinking water impacts sanitation, where infectious diseases can develop and spread [3,17]. This makes it difficult to access essential maternal and child health services timely and affordable [14,15,18]. Furthermore, inadequate WASH services in healthcare facilities have also been associated with increased patient dissatisfaction and even a barrier to service utilization in some settings (including maternity services) [3,13,18]. Indeed, the lack of high-quality WASH facilities in delivery rooms is often cited as why women prefer home delivery [3,18].

Women expect health facilities to have an adequate WASH system, essential for their human rights, dignity, and infection prevention. However, achieving this goal remains a distant prospect in many health facilities in developing countries [18]. Satisfaction with health services is defined as the extent to which patients positively perceive the care nurses or medical staff provide [19]. Patient satisfaction reveals the time to which healthcare needs are being met and provides a key indicator of high-quality healthcare. In addition, it is a factor used for planning and evaluating health interventions [19]. Numerous studies have shown that patients satisfied with the care provided by health personnel are more likely to use health services in the future and to comply with the prescribed medical treatment until the end. Therefore, for patients to be more satisfied with the treatment, there is a need to provide high-quality healthcare considered safe, timely, effective, efficient, equitable, and patient-centred [18,19]. Providing high-quality care in maternity services involves giving mothers the best possible medical care and outcomes during the antenatal, delivery, and postnatal periods that can be measured against standard guidelines [20]. Evidence shows that pregnant women are more likely to give birth in health facilities if satisfied with their care at service delivery points [21-23]. However, other studies have shown that poor quality of services and negative attitudes of health workers prevent pregnant women from using these services [21,24]. Therefore, in addition to improving facility infrastructure, quality of care, and cost-effectiveness, improvements in maternity services should also address providers' interpersonal attitudes and behaviors [24,25]. It was relevant to have data on the perceptions and experiences of the key stakeholders to demonstrate that the effects of the BECEYA project go beyond improving the health environment.

The results also show that the BECEYA project has brought about profound changes in health facilities. These changes have positively influenced female service users' motivation to attend health centers and improved the quality of care. However, despite the significant effects of the BECEYA project, structural barriers prevent all women from benefiting from the best pregnancy care. One of these major barriers is the need for more financial resources to meet the cost of the services [25]. Indeed, implementing strategies designed to encourage attendance at health centers for antenatal care worldwide tends to assume that people will attend if there is a good quality of services. However, sociodemographic data indicate that women from relatively poor backgrounds, living in rural areas and/or with low levels of education, are less likely to access antenatal services, even if available [26]. Infrastructures must be maintained and upkeep to ensure the sustainability of these interventions. The ongoing staff training also needs to be addressed [3]. This study has some limitations. The choice of the two health centers studied was not made randomly, and the perceptions analyzed only reflect the realities of these two contexts. Furthermore, to please, the participants may have responded according to social desirability.

## Conclusion

This study aimed to evaluate the intervention's effects on improving the health environment for mothers and children in health centers. The study highlighted some positive impacts on women users, their companions, and health center staff following the implementation of the intervention. These changes have had positive results for women attending antenatal care and their companions' (better care experience, improved quality of care) and health center staff (improved environment and working conditions). This study contributes to showing some links between improving the environment of healthcare centers and the quality of care in developing countries.

### What is known about this topic

- *Essential water, sanitation, and hygiene services in healthcare facilities are fundamental to providing quality;*
- *In healthcare facilities, WASH interventions promote a healthy environment to minimize disease risk for users and the surrounding community;*
- *Sub-Saharan Africa has the lowest access to safe water and sanitation initiatives and the lowest rate of improvement in sanitation*.

### What this study adds

- *Improvements in the healthcare environment were associated with an increase in the quality of services and work capacity;*
- *The intervention resulted in an unprecedented motivation among women to use health services and their commitment to maintaining cleanliness in their living environment;*
- *This study contributes to showing some links between improving the environment of healthcare centres and the quality of care in developing countries*.
